# Longitudinal age-dependent effect on systolic blood pressure

**DOI:** 10.1186/1753-6561-3-s7-s87

**Published:** 2009-12-15

**Authors:** Bonnie R Joubert, Guoqing Diao, Danyu Lin, Kari E North, Nora Franceschini

**Affiliations:** 1 Department of Epidemiology, Gillings School of Global Public Health, University of North Carolina, 2101 McGavran-Greenberg Hall, CB #7435, Chapel Hill, North Carolina 27599, USA; 2 Department of Statistics, George Mason University, 4400 University Drive, Fairfax, Virginia 22030, USA; 3 Department of Biostatistics, Gillings School of Global Public Health, University of North Carolina, 2101 McGavran-Greenberg Hall, CB #7420, Chapel Hill, North Carolina 27599, USA; 4 Carolina Center for Genome Sciences, University of North Carolina, 5009 Genetic Medicine Building, Chapel Hill, North Carolina 27599, USA

## Abstract

Age-dependent genetic effects on susceptibility to hypertension have been documented. We present a novel variance-component method for the estimation of age-dependent genetic effects on longitudinal systolic blood pressure using 57,827 Affymetrix single-nucleotide polymorphisms (SNPs) on chromosomes 17-22 genotyped in 2,475 members of the Offspring Cohort of the Framingham Heart Study. We used the likelihood-ratio test statistic to test the main genetic effect, genotype-by-age interaction, and simultaneously, main genetic effect and genotype-by-age interactions (2 degrees of freedom (df) test) for each SNP. Applying Bonferroni correction, three SNPs were significantly associated with longitudinal blood pressure in the analysis of main genetic effects or in combined 2-df analyses. For the associations detected using the simultaneous 2-df test, neither main effects nor genotype-by-age interaction *p*-values reached genome-wide statistical significance. The value of the 2-df test for screening genetic interaction effects could not be established in this study.

## Background

Systolic blood pressure (SBP) increases with age due to increased peripheral vascular resistance, which accounts for increased prevalence of hypertension after age 50 [1]. Age-related effects on SBP may result from genetic effects or from environmental effects (for example, dietary salt intake) in the context of genetic susceptibility to hypertension [2,3]. Failure to account for genotype-by-age interaction may prevent the identification of genetic variants influencing blood pressure variation and hypertension susceptibility in populations.

In this paper, we examined the effect of genotype-by-age interaction on SBP. To this end, we present a novel variance-component method for the estimation of age-dependent effects on SBP using the longitudinal SBP data and a subset of the Affymetrix 500 k single-nucleotide polymorphisms (SNPs) genotyped in the Framingham Heart Study (FHS) Offspring Cohort.

## Methods

### Population, study design, and phenotypes

The FHS was started in 1948 as a community-based cohort study of cardiovascular disease. In 1971, 5,124 subjects were enrolled in the FHS Offspring Cohort. These individuals were children and spouses of the children of the Original Cohort. The FHS Offspring Cohort were followed prospectively, and examined during four clinical visits [4]. The FHS Offspring Study protocol was reviewed by the Boston University Medical Center Institutional Review Board and all participants gave informed consent [5]. Our study included all offspring participants whose phenotype and genotype data were available through the Genetic Analyses Workshop 16, Problem 2. We excluded twins (*n *= 10), individuals on hypertension treatment or unknown treatment at Visit 1 (*n *= 64), and those individuals with missing SBP measurements at Visit 1 (*n *= 106). The study was conducted in accordance with the principles of the Declaration of Helsinki.

We analyzed genotype-by-age interaction for longitudinal SBP. After being in a seated position for at least 5 minutes, each subject was fitted on the left arm with a mercury sphygmomanometer and a cuff long enough to fit the most obese arm. A physician then measured SBP. SBP measures taken during four consecutive clinical visits were used for analysis.

### Genotyping and quality control

SNPs were genotyped on the Affymetrix GeneChip Human Mapping 500 k Array Set. Genotyping calls were made with the BRLMM (Bayesian robust linear model with Mahalanobis distance classifier) algorithm. The FHS applied a minimum set of SNP quality control filters by removing individuals that incorrectly identified their sex and removing SNPs with Mendelian inconsistencies. We also filtered for SNPs not mapped to a chromosome (chromosome 0) and those not located in autosomal chromosomes (*n *= 12,398), duplicate SNPs (removed those with missing position, *n *= 1,132), SNPs with Mendelian inconsistencies, and SNPs with minor allele frequency (MAF) < 0.01.

### Statistical analyses

We developed a powerful and robust variance-component method to examine the association between a quantitative trait and SNPs in the context of longitudinal data. Our method is based on semiparametric transformation linear models, which allow arbitrarily distributed quantitative traits [6]. In simulation studies, this approach is robust to nonnormality and outliers and performs as well as parametric methods when the normality assumption is satisfied. In the presence of non-normality, our approach is substantially more powerful than its counterpart with parametric transformations [7].

We examined genotype-by-age interaction on SBP using the Offspring FHS data, while allowing for family structure and the longitudinal cohort component of the data. Models were adjusted for sex. Specifically, we implemented the following model:

where H is an unknown increasing function and can be consistently estimated from the data; β_1_, β_2_, β_3_, and β_4 _are the sex effect, age effect, main genetic effect, and genotype-by-age interaction, respectively; *a *is a random intercept accounting for the correlations of the repeated measurements from the same individual; *g *is a polygenic random effect accounting for the correlations due to common genetic factors; and *e *is an individual-specific residual error. The random effects are assumed to be normally distributed. This model consists of two parts, of which the mean part models the effects of covariates and the association between SNP genotype and longitudinal quantitative traits and their interactions, and the variance-component part models the correlations among trait values within a family as well as intra-subject correlation among multiple measurements per subject. It is straightforward to include random slope in the above model. Hypothesis testing was performed using likelihood-ratio test statistics (LRT). The following models were tested:

Model 1: *E *[*H*(*Y*)] = β_1_(*sex*) + β_2_(*age*)

Model 2: *E *[*H*(*Y*)] = β_1_(*sex*) + β_2_(*age*) + β_3_(*SNP*)

Model 3 (Full Model): *E *[*H*(*Y*)] = β_1_(*sex*) + β_2_(*age*) + β_3_(*SNP*) + β_4_(age*SNP),

where *Y *is the longitudinal SBP.

(A1) LRT1: Testing H_0_: β_3 _= 0 vs. H_A_: β_3_≠0, compares Model 2 with Model 1, 1 df

(A2) LRT3: Testing H_0_: β_4 _= 0 vs. H_A_: β_4_≠0, compares Model 3 with Model 2, 1 df

(A3) LRT3: Testing H_0_: β_3 _= β_4 _= 0 vs. H_A_: β_3_≠0 or β_4_≠0, compares Model 3 with Model 1, 2 df.

The corresponding null distributions of the test statistics for (A1), (A2), and (A3) are approximately chi-square with 1 df, 1 df, and 2 df, respectively.

## Results

A total of 2,475 Offspring Cohort participants were available for analysis. In order to preserve computational resources, analyses were completed for chromosomes 17-22 only. This subset was judged to provide sufficient data for a comparison of genome-wide statistical significance across three models of interest, but not sufficient for retrieval of novel variants. Before quality control filtering, a total of 58,033 SNPs were available, including 11,232 for chromosome 17, 14,832 for chromosome 18, 6,350 for chromosome 19, 12,367 for chromosome 20, 7,085 for chromosome 21, and 6,167 for chromosome 22. After implementing quality control filters, a total of 57,827 SNPs remained for analyses: 11,176 for chromosome 17, 14,818 for chromosome 18, 6,262 for chromosome 19, 12,352 for chromosome 20, 7,078 for chromosome 21, and 6,141 for chromosome 22. FHS participants were on average 33 years old (SD = 9) at Visit 1 and 54% were women. The mean SBP at first visit was 119 mm Hg (SD = 14). At the last visit, the mean age was 60 years (SD = 9) and the mean SBP was 126 mm Hg (SD = 19).

**Figure 1:**
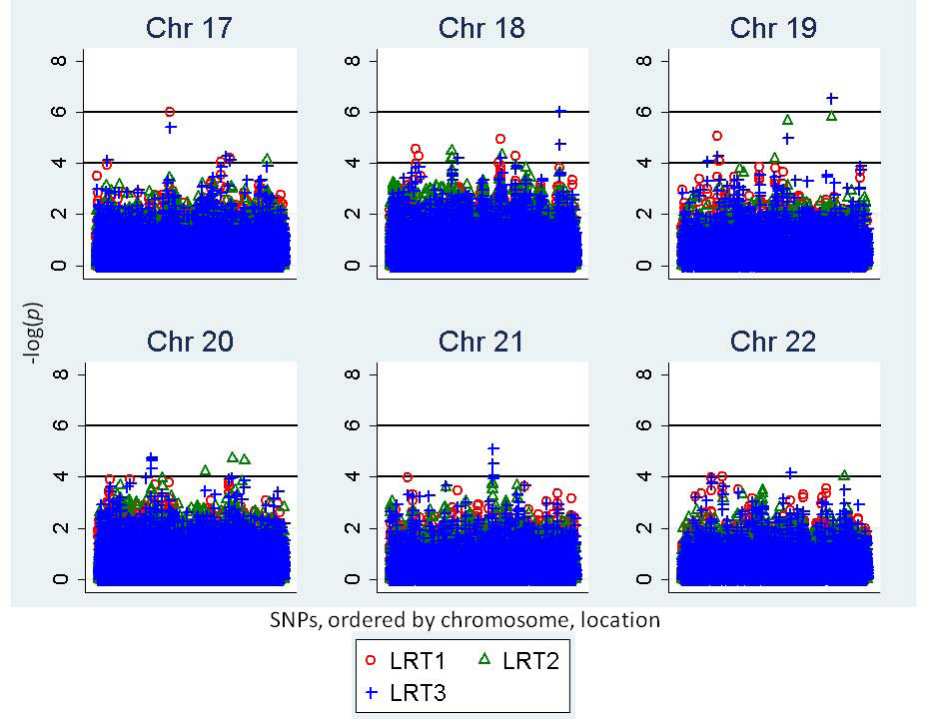
**LRT -log(*p*) values for the association between SNPs and longitudinal systolic blood pressure, for the main genetic effect (LRT1), genotype-by-age interaction (LRT2), and combined main genetic effects and genotype-by-age interaction (LRT3)**. Results for all SNPs on chromosomes 17-22 are displayed.

Table 1 and Figure 1 display the results of analysis for the main genetic effect (LRT1), genotype-by-age interaction (LRT2), and combined analysis of main genetic and genotype-by-age interaction (LRT3) on longitudinal SBP. Three SNPs reached genome-wide statistical significance for association with longitudinal SBP using a *p*-value of 8.64 × 10^-7 ^(Bonferroni correction 0.05/57,827 tests, negative log base 10 *p*-value (-log(*p*)) = 6.0). If additional correction for the three models was performed, only one SNP association reached statistical significance (*p*-value = 2.88 × 10^-7^, -log(*p*) = 6.54).

**Table 1:** LRT and *p*-values for the association between SNPs and longitudinal SBP for the main genetic effect (LRT1), genotype-by-age interaction (LRT2), and combined main effects and genotype-by-age interaction (LRT3)

					Genetic effect		Genotype-by-age effect		Genetic + genotype-by-age effects	
							
SNP^a^	Chr^b^	Position	Alleles	MAF	LRT1	-log(*p*)	LRT2	-log(*p*)	LRT3	**-log(*p***)
rs17631940	17	30842243	A/G	0.49	23.99	6.01	1.02	0.50	25.00	5.43
rs1476112	18	170690	T/G	0.08	14.40	3.83	13.43	3.61	27.83	6.04
rs8102150	19	922139	G/C	0.03	7.05	2.10	23.09	5.81	30.14	6.55

^a^Only SNPs with a Bonferroni-corrected *p*-value ≤ 10^-7 ^(-log(*p*) ≥ 6) in at least one of the tests are shown.

^b^Chr, chromosome; MAF, minor allele frequency

Of the three SNPs with a -log(*p*) ≥ 6, one signal corresponded to the main genetic effects, and the remaining two signals were for the 2-df test, which simultaneously estimated the main genetic effect and genotype-by-age interactions for each SNP. None of the SNPs obtained a -log(*p*) ≥ 6 for the 1-df genotype-by-age analysis.

## Discussion

In this paper, we applied a novel variance-component method for the estimation of age-dependent effects on a quantitative character (SBP) in the context of Affymetrix 500 k SNPs genotyped in the FHS Offspring Cohort. Differences in the expression of SBP or differences in genes contributing to the variation in susceptibility to hypertension by age are plausible and supported by several lines of evidence [1,2,8]. First, many studies have suggested differences in risk of hypertension by age [1,2]. In addition, several studies have reported associations of blood pressure related phenotypes with candidate gene polymorphisms or candidate regions that are modified by age [8-10]. We interrogated the effect of genotype-by-age interaction on SBP but did not find evidence of significant interaction in the subset of SNPs in the sampled chromosomes that were evaluated.

We were able to detect significant main genetic effects for one SNP, rs17631940, located at the 17q12 region, in the intron 2 of the schlafen family member 12-like (*SLFN12L*) gene (Table 1). The *SLFN12L *gene product is involved in cell growth and T cell development [11], and the gene's association with blood pressure phenotypes has not been previously reported. Interestingly, two significant genome-wide associations were identified using the 2-df test, but none of the SNPs were significantly associated in analysis of main genetic effects or in genotype-by-age interaction. These SNPs were rs1476112, located on the *DKFZP781G0119 *gene (18q22.3 region), and rs8102150, located at the 19q13.33 region and not within a gene. These findings are intriguing and suggest that genes may influence a trait by a combination of main and interacting effects. However, these findings will need to be replicated to distinguish this signal from a false-positive finding. In addition, the utility of 2-df test for screening genetic interaction effects could not be established in this study.

## Conclusion

We did not identify SNPs with specific genotype-by-age interaction effects on SBP. Accounting for interaction with environmental factors such as age may improve our ability to detect genetic effects, but some of the effects may result from a combination of main and environment interacting genetic effects on phenotypes. The identification of genetic factors, both in the absence of and in combination with age effects, that influence the risk of hypertension, can provide new insights into susceptibility and contribute important new information for understanding the mechanistic basis of cardiovascular disease.

## List of abbreviations used

FHS: Framingham Heart Study; LRT: Likelihood-ratio test; MAF: Minor allele frequency; SBP: Systolic blood pressure; SNP: Single-nucleotide polymorphism.

## Competing interests

The authors declare that they have no competing interests.

## Authors' contributions

NF, DL, and KEN are responsible for the study design. NF, DL, KEN, and BRJ are responsible for writing the manuscript. BRJ, NF, and GD performed the analysis. All authors reviewed the manuscript.
